# Alpha-gal allergy after a tick bite in Austria

**DOI:** 10.1007/s00508-019-1506-5

**Published:** 2019-05-13

**Authors:** Veronika Pisazka, Georg Duscher, Adnan Hodžić, Norbert Reider, Franz Allerberger

**Affiliations:** 1 Austrian Agency for Health and Food Safety (AGES), Spargelfeldstraße 191, 1220 Vienna, Austria; 2 Institute of Parasitology, University of Veterinary Medicine Vienna, Vienna, Austria; 3 Department of Dermatology, Venereology and Allergy, Medical University Innsbruck, Innsbruck, Austria

**Keywords:** Alpha-gal syndrome, Ixodes ricinus, Tick bites, Meat allergy, Anaphylaxis

## Abstract

Tick bites can cause the alpha-gal syndrome, which is characterized by delayed anaphylactic reactions mainly to red meat and offal due to IgE antibodies against mammalian galactose-alpha-1.3-galactose carbohydrate (alpha-gal). *Ixodes ricinus *bites are considered the primary cause of IgE antibody responses specific for alpha-gal in Europe. This article reports on a 51-year-old Austrian male who acquired a tick bite in Austria in spring 2017, which, within 48 h, resulted in prolonged inflammation of the skin area around the bite. The patient experienced an allergic reaction 3 months later approximately 8 h after eating a medium rare steak for dinner. The symptoms included an itchy rash on both sides of the torso and on both arms which persisted for several hours. In spring 2018, the patient suffered another tick bite. The patient’s skin reaction was similar to that of the previous year. In the following months, the patient experienced five episodes of severe allergic reactions, each during the night after having eaten beef for dinner. The symptoms included pruritic urticarial rash involving the entire body along with swollen hands, diarrhea, vomiting and in some episodes even shortness of breath. At the request of the patient, specific IgE antibodies against alpha-gal were determined, revealing a highly positive result (>100 kU/l). This brief report aims to raise awareness that recurrent delayed anaphylactic reactions to food can develop after tick bites.

In 2007, O’Neil et al. reported a hitherto unknown high rate of anaphylactic reactions in patients treated with infusions of cetuximab, an antibody against the epidermal growth factor receptor, in south-eastern regions of the USA [1] and 1 year later, Chung et al. identified IgE antibodies specific for galactose-alpha-1.3-galactose (alpha-gal), an oligosaccharide present on the antigen-binding (Fab) fragment of the cetuximab heavy chain portion, as the underlying cause [2]. In 2009, Van Nunen et al. described an association between severe local reactions to tick bites and anaphylaxis to red meat in Australia [3]. At the same time Commins et al. identified IgE to alpha-gal in a group of patients with similar reactions to red meat in the USA [4]. Further investigations demonstrated the presence of alpha-gal in the gastrointestinal tract of the tick *Ixodes ricinus* [5]. A recent study demonstrated its involvement in tick-pathogen interactions and possibly in the development of alpha-gal allergy [6].

The alpha-gal epitope is expressed on cells of non-primate mammals whereas humans, apes and Old World monkeys lack the synthetic machinery to generate the alpha-gal, which can lead to a strong immune response against the glycan molecule [7]. Bites by lone star ticks (*Amblyomma americanum*), prevalent in the south-eastern regions of the USA, are considered to be the primary cause of IgE antibody responses specific for alpha-gal in the USA [8]. In recent years, the alpha-gal syndrome has been diagnosed on several continents, including Europe [9–13]. In southern Sweden, the alpha-gal syndrome has been related to bites of *Ixodes ricinus *[9], which is also the most dominant tick species in Austria.

Patients with alpha-gal-mediated delayed meat allergy display enhanced alpha-gal-specific IgE levels accompanied by high levels of alpha-gal-specific IgG1, the latter being atypical for food allergies and distinct from natural alpha-gal responses in individuals allergic to substances other than meat [14]. Nevertheless, the syndrome is still relatively unknown in primary care settings and due to the atypical delayed onset of allergic symptoms, usually 3–7 h after the ingestion of mammalian food products, the syndrome often remains undiagnosed. This article reports on a 51-year-old Austrian male with alpha-gal syndrome, whose diagnosis was patient-driven, in order to raise awareness for this rare type of allergy. The patient gave written informed consent for publication of the clinical details. A 51-year-old sportsman (blood group A, rhesus positive), with a known history of cat allergy, acquired a tick bite in Lower Austria, an eastern federal state of Austria, in spring 2017. In the province of Lower Austria, *Ixodes ricinus* accounts for approximately 95% of all ticks detectable on hosts. This resulted in inflammation of the skin around the bite within 48 h after exposure. Symptoms included swelling, redness and itchiness and persisted “for a few weeks”. The patient was tested for antibodies directed against *Borrelia*, yielding a negative result but 3 months later, the patient experienced an allergic reaction approximately 8 h after eating a medium rare steak for dinner. The symptoms included an itchy rash on both sides of the torso and on both arms, which persisted for several hours. A primary care physician diagnosed an allergic exanthema possibly of food-borne origin. In spring 2018, the patient acquired another tick bite, again in the same eastern region of Austria. The patient’s skin reaction was similar to that of the previous year. In the following summer months, the patient experienced five episodes of severe allergic reactions, each during the night after having eaten beef for dinner. The symptoms included pruritic urticarial rash involving the entire body along with swollen hands, diarrhea, vomiting and in some episodes even shortness of breath. In each episode, the symptoms resolved after several hours without medical treatment. After these reactions, the patient started to avoid food containing beef. In late summer 2018, the patient consumed a meal of suckling pig for lunch and again reacted with pruritic urticarial rash, in this case with onset of symptoms approximately 2.5–3 h after the meal. A primary care physician administered cortisone and antihistamine intravenously and the symptoms resolved within a few hours. On request of the patient, an alpha-gal test was performed in an outpatient department specialized in allergies. Laboratory testing (ImmunoCAP^TM^, Thermo Fisher Scientific, Vienna, Austria) revealed a high titer of IgE antibodies against alpha-gal (>100 kU/l), pork (3.54 kU/l) and beef (11.10 kU/l), confirmed the history of cat epithelia allergy (3.32 kU/l) and revealed the presence of antibodies against the major cat allergen Fel d 1 (3.59 kU/l); normal values for specific IgE: <0.35 kU/l, normal range for total IgE ≤100 kU/l (Table 1). Results for cow milk allergens Bos d 4, 5, 6, and 8 were negative.

**Table 1:** IgE levels to alpha-gal, pork, beef, cat epithelia, Fel d 1 (the major allergen of domestic cats), Fel d 2 (cat serum albumin), and to house dust mites (*Dermatophagoides pteronyssinus*), in an Austrian patient with delayed meat allergy

Allergen	IgE kU/l	CAP class
Total IgE	752 (≤100)	–
Alpha-gal	>100	6
Pork	3.54	3
Beef	11.10	3
Cat epithelia	3.32	2
Fel d 1	3.59	3
Fel d 2	0.06	0
House dust mites (*Dermatophagoides pteronyssinus*)	19.6	4

*kU/l* kilounits per liter, *CAP class* ImmunoCAP^TM^ quantitative scoring guide

In addition to measuring IgE titers, a skin-prick test was performed which showed a strong reaction to cat epithelia but interestingly no reaction to beef, pork and lamb. Egg white, chicken and codfish were also negative. Based on these results, the diagnosis of alpha-gal allergy was made and the patient was prescribed an emergency kit including epinephrine auto-injector (EpiPen®, Meda Pharma, Vienna, Austria). Additionally, he was advised to avoid mammalian meat products (e.g. beef, pork, lamb and venison) and offal (e.g. kidneys) and to be cautious with mammalian products containing gelatine (e.g. desserts, fruit gums and medicinal intravenous infusions). The patient tolerates fish and poultry well without any allergic reactions or symptoms.

As discussed above, the high prevalence of rapid and occasionally fatal hypersensitivity reactions following or during the first infusion of cetuximab, an oncologically applied chimeric mouse-human IgG1 monoclonal antibody against the epidermal growth factor receptor, with alpha-gal on both of its Fab segments, in some areas of the USA in 2004–2008 [2, 15], finally led to the identification of a novel form of food allergy, the so-called alpha-gal syndrome [3].

Due to the fact that, in contrast to non-primate mammals, humans, apes and Old World monkeys lack the synthetic machinery to generate alpha-gal, all non-immunocompromized humans produce abundant natural antibodies to alpha-gal, predominantly IgM and IgG2, which represent the dominant mechanism of hyperacute rejection in porcine xenotransplantation [16]. Distinct from natural alpha-gal responses in individuals allergic to substances other than meat, those with alpha-gal syndrome display enhanced alpha-gal-specific IgE levels accompanied by high levels of alpha-gal-specific IgG1 [14]. Patients with delayed meat allergy display IgE and IgG antibodies that selectively recognize the alpha-gal epitope on bovine gamma globulin; poultry and fish do not express alpha-gal [14].

The patient had a history of strong and prolonged local inflammatory reactions to tick bites. As described in the literature [3, 8], this can be a hint for sensitization to alpha-gal. No such local inflammatory reactions had occurred after the few tick bites the patient had experienced in previous years. This also underlines that only very few tick bites can be sufficient to induce the alpha-gal syndrome. The patient had a manifest cat allergy. It has been shown that a high proportion of alpha-gal positive subjects react to cat epithelia extracts in vitro, without a history of clinical allergy to cats. These subjects, however, do not react to the major cat allergen Fel d 1, found in cat hair, dander and saliva but to the cat IgA Fel d 5, which cross-reacts with alpha-gal [17]. Unfortunately, Fel d 5 is not commercially available for testing. Rare cases of immediate, not delayed, anaphylactic reactions to pork have been reported in patients allergic to cats. This is due to a sensitization against Fel d 2, the cat serum albumin. In the present case, the patient proved to be sensitized to Fel d 1 but not to Fel d 2. Thus, the cat and his pork allergies must be regarded as independent from each other.

Studying red meat allergy in Sweden, Hamsten et al. identified an association with tick sensitization and B‑negative blood groups: of 39 patients they studied, all but 2 belonged to the blood groups A or O, which is significantly more compared with the expected number in the Swedish population (82%) [9]. That red meat allergy is strongly associated with the B‑negative blood groups, can be explained by the fact that the alpha-gal epitope, a major blood group substance of nonprimate mammals, is structurally related to blood group B [9]. The present patient showed blood group A, rhesus positive.

The reason why alpha-gal allergic subjects do not always have an allergic reaction to meat and if they do react, show symptoms only several hours after the consuming the food, has not been fully elucidated. It has been hypothesized that cofactors, such as physical exercise or concurrent intake of alcohol or non-steroidal anti-inflammatory drugs may play a role. Moreover, alpha-gal as a component of glycoproteins and glycolipids, is resorbed in chylomicrons and very low-density lipoproteins (VLDL) which are resorbed more slowly than, e.g. glucose in the gastrointestinal tract [18].

Allergy to alpha-gal can elicit potentially life-threatening symptoms including anaphylactic shock. It has a substantial impact on the life style habits of those persons affected. Although the syndrome has been recognized on several continents during the past years, the delayed onset of symptoms 3–7 h after ingestion of mammalian meat food products and the fact that the symptoms do not occur with every exposure to red meat in patients, who had tolerated meat for many years previously, often makes it difficult for patients and their health care providers to identify the causal antigen. Diagnosis of this allergy relies on measuring serum IgE-antibody titers against the alpha-gal carbohydrate in combination with the patient’s history of delayed allergic reactions after the ingestion of mammalian food products. Skin-prick tests are of limited benefit and depend on the extracts used [18]. Patients have to avoid red meat, mammalian products containing gelatine [19, 20] and in some cases even dairy products as the carbohydrate moiety alpha-gal is found in mammalian milk [21]. Also, infusions containing gelatine should be avoided in any treatment of these patients. Anaphylactic reactions to porcine kidney in France, Germany and Luxembourg has also been attributed to alpha-gal-specific IgE antibodies [18, 22, 23]. For the management of severe acute reactions, emergency kits including epinephrine autoinjectors (EpiPen®) are handed out to individuals with diagnosed alpha-gal syndrome.

Kollmann et al. recently reported on 20 patients who had presented with severe, delayed hypersensitivity reactions on consuming red meat at Austrian allergy clinics over a period of 10 years, all of them displaying IgE and IgG antibodies that selectively recognized the alpha-gal epitope on bovine gamma globulin [14]. This short report aims to raise awareness of this form of alpha-gal-mediated delayed meat allergy, atypical for food allergies, among physicians not specializing in allergies.
